# PEG4000 modified liposomes enhance the solubility of quercetin and improve the liposome functionality: in vitro characterization and the cellular efficacy

**DOI:** 10.55730/1300-0527.3411

**Published:** 2022-02-23

**Authors:** Gülen Melike DEMİRBOLAT, Ömer ERDOĞAN, Göknil Pelin COŞKUN, Özge ÇEVİK

**Affiliations:** 1 Department of Pharmaceutical Technology, Faculty of Pharmacy, Acıbadem Mehmet Ali Aydınlar University, İstanbul, Turkey; 2 Department of Biochemistry, School of Medicine, Aydın Adnan Menderes University, Aydın, Turkey; 3 Department of Pharmaceutical Chemistry, Faculty of Pharmacy, Acıbadem Mehmet Ali Aydınlar University, İstanbul, Turkey

**Keywords:** Quercetin, solubility, PEGylated liposomes, drug delivery, antitumoral effect, mitochondrial apoptosis

## Abstract

Quercetin, a multifunctional therapeutic agent, is used in various types of cancer. However, its therapeutic effect is limited by virtue of poorly aqueous solubility and instability in the physiological medium. To overcome these limitations, we aimed (i) to design quercetin loaded liposomes with unlinked-PEG4000 with regard to not only surface modification but also solubility enhancement, and (ii) to investigate the antineoplastic effects on HeLa cells. PEG4000 increased the quercetin solubility 2.2 fold. PEG4000 modified liposomes displayed small particle size (254 ± 69 nm), low polydispersity index (0.236 ± 0.018), favorable zeta potential (–35.4 ± 0.6 mV), high quercetin encapsulation efficiency (87.6 ± 5.6%), and drug loading (22.2 ± 6.9%). The homogeneity and particle size of stable PEGylated liposomes were proved by transmission electron microscopy. The drug release was reached up to 65.1 ± 3.8% in 6 h. The IC_50_ value of quercetin loaded PEGylated liposomes was 16.3 μg/mL on HeLa cells, while that of quercetin was 88.3 μg/mL. PEGylated liposomes remarkably hampered the adherence and colony formation ability of cells according to crystal violet staining tests. The convenience of PEGylated liposomes for the parenteral application was stated by the hemolysis assay. The high-throughput screening assays based on AO/PI staining proved the drastic decrease of viable cell count. Moreover, qPCR tests based on gene expression levels revealed that the quercetin loaded PEGylated liposomes treatment could be more effective than free quercetin on the mitochondrial apoptosis of HeLa cells. These promising results allow considering further in vivo studies for efficient cancer treatment with quercetin loaded PEG4000 modified liposomes.

## 1. Introduction

Quercetin (QC) is a versatile therapeutic agent with antioxidant, antihypertensive, antithrombotic, angioprotective, and antiinflammatory effects, and also used for preventing obesity-related diseases, as well as to treat some kinds of cancer [1]. By having these properties, it has gained widespread attention for various potential applications in the healthcare industry. However, its poorly aqueous solubility, instability in the physiological medium, and low bioavailability remain as main obstacles in using QC in the pharmaceutical field [2]. The water solubility of quercetin is quite low, 1.53–12.5 mg/L at gastrointestinal pH levels (pH 2–7) [3], and so there have been many attempts to alleviate this fundamental problem with the help of cosolvent effect, surfactant effect, inclusion complex or nanosized drug delivery systems. In one of these applications, DMSO was used to dissolve QC, but such concerns as the requirement of higher doses, the occurrence of dose-dependent hemolysis, and unpleasant odor hampered the DMSO usage [4]. Apart from DMSO, adding propylene glycol into the solvent systems also increased the solubility of QC [5]. Cosolvent effects might enhance the solubility, but not alter the instability of QC in the physiological medium. Therefore, developing the new dosage forms of QC with increased solubility and improved in vivo stability is highly needed. In this particular case, the importance of QC encapsulation cannot be neglected. It was reported that nanoencapsulation enhances the solubility and pharmacokinetics profiles of insoluble drugs [6]. Thus, extensive efforts related to inclusion complexes, prodrugs, nanocrystals, polymeric nanoparticles, microparticulates, magnetic nanoparticles have been made [7–11]. Among drug carrier systems, lipid-based systems take a step forward since the presence of fat in formulation increases the QC bioavailability [5, 12].

Liposomes which are the lipid-based systems are selfassembled lipid bilayer structures with phospholipid and cholesterol components. The size of these nearly spherical lipid vesicles can usually range between 50 and 450 nm for medical application [13]. Liposomes have good compatibility because they are nontoxic, nonimmunogenic, and completely biodegradable. They increase efficacy, stability, and function for a long time and reduce the encapsulated agent’s toxicity [14]. The physiobiochemical characteristics of liposomes can be tailored by altering the types and ratios of component and decorating the liposomal surface with biocompatible hydrophilic polymers like polyethylene glycol (PEG) [15]. Using PEGs with different molecular weights on liposome surface, also known as PEGylation, provides a shielding effect for enzymes to access lipid vesicles due to stealth properties of PEG chain [16]. These modifications can give drastic effects especially on biological environment as liposomes recognition of opsonins and liposomal clearance by the mononuclear phagocyte system slow down and liposome circulation time extends [17,18]. In briefly, PEGylation positively affects on the stability, passive targeting ability to tumoral tissues, circulation time, therapeutic effects and reduction of the toxicity of encapsulated drug [19]. Therefore, the entrapment of hydrophobic QC in these stealth liposomes can be a feasible strategy to overcome its poor solubility in aqueous medium, in other terms, the main challenges of QC formulation.

In this study, we developed QC-loaded PEGylated liposomes and explored the effect of their apoptotic activity in cell cultures, comparing to QC-loaded unmodified liposomes. Stable QC loaded stealth liposomes with small size and high encapsulation efficiency were suitably produced for parenteral administration. Even when used at minimal doses, they augmented their anticancer efficacy on cervical cancer cells in cell viability, colony formation, cell staining and gene expression.

## 2. Materials and methods

### 2.1. Materials

Quercetin (≥95% HPLC), L-α-phosphatidylcholine (≥99%, egg yolk, lyophilized powder), cholesterol, PEG2000, and PEG4000 were purchased from Sigma-Aldrich (St. Louis, MO, USA). Phosphate buffered saline (PBS) tablets were obtained from BioShop (Ontario, Canada). The cervical cancer cells (HeLa) were obtained from ATCC (Manassas, VA, USA). Dulbecco’s modified Eagle’s medium (DMEM), MTT (3-(4,5-dimethylthiazol-2-yl)-2,5-diphenyltetrazolium bromide), Bax, Bcl-2, and GAPDH primers were purchased from Invitrogen (Thermo Fisher Scientific, USA). All other reagents were of analytical grade.

### 2.2. Solubility of QC

The solubility of QC was evaluated in distilled water, in PEG2000 aqueous solution and in PEG4000 aqueous solution to investigate the effect of molecular weight of PEG as a surface coating material on the solubility of QC. To this end, an excess amount of QC was added to 10 mL of distilled water and mixed with PEG2000 or PEG4000. Then, the mixtures were stirred at 1000 rpm using a magnetic stirrer for 48 h. Then, the dispersion was filtered through a membrane filter (0.22 μm). The completely dissolved amount of QC was analyzed via double beam UV-VIS spectrophotometer (Shanghai Mapada Instruments UV-6100, China) [20,21]. Briefly, certain amount of QC was dissolved in methanol, and a series of QC samples at different concentrations were analyzed. The linear calibration curve was obtained in the range of 0.2–14 μg/mL for QC.

### 2.3. Preparation of liposomes

PEGylated liposomes encapsulating quercetin were prepared by thin film hydration method [4]. L-α-phosphatidylcholine, cholesterol, quercetin and PEG4000 were dissolved in chloroform and methanol (3:1, v/v) in a round bottom flask. The organic solvent was evaporated under reduced pressure at 50 rpm for 4 h at 60 °C on a rotary evaporator (Buchi Rotavapor R100). After the obtained thin film was remained overnight in a vacuum oven at room temperature for removal of residual organic solvent, it was rehydrated 10 mL of phosphate buffer saline (PBS, pH: 7.4). The mixture was subsequently vortexed for 5 min and sonicated for 10 min. The liposomes were subjected to the centrifugation at 14,000 g for 1 h to remove any unentrapped quercetin. The precipitates were suspended with PBS. Other produced liposomes (LQ1: quercetin loaded non-PEGylated liposomes; L1 and L2; non-PEGylated and PEGylated quercetin-free formulations, respectively) were prepared following the same procedure without using quercetin and/or PEG in the process. The compositions of liposomes were given in Table.

### 2.4. Characterization of liposomes

#### 2.4.1. Particle size and zeta potential measurements

The particle size (PS), and polydispersity index (PDI) of liposomes were measured by dynamic light scattering (DLS) using Litesizer 100 (Anton Paar, Austria). The scattering data were recorded at a 90° angle. The zeta potential (ZP) of them were measured with the same device by applying laser Doppler velocimetry (LDV). The dispersions were diluted more than a hundred times with PBS and analyzed six times.

#### 2.4.2. The morphological investigation

QC loaded liposomes (LQ1 and LQ2) were visualized with Thermo Scientific Talos L120C transmission electron microscope (Thermo Fisher Scientific, Massachusetts, USA). A sufficient quantity of samples was deposited on a carbon coated TEM copper grid and allow to dry in air at room temperature to form thin films. TEM images were taken at suitable magnifications after the grid insertion in TEM.

#### 2.4.3. Encapsulation efficiency and drug loading capacity

The amount of quercetin entrapped in liposomes was determined through the indirect method based on the measurement of the drug present in the external phase [22]. After the centrifugation process in the liposomes preparation, the supernatant containing free drug was analyzed for the drug content via double beam UV-VIS spectrophotometer (Shanghai Mapada Instruments UV-6100, China) at 268 nm and 375 nm where the quercetin exhibits two major absorption peaks [23]. Although the absorbance values at both wavelengths were recorded, data collected at 268 nm was used in all calculations due to high consistency. The amount of QC was calculated using the previously subtracting calibration curve. The percentage of drug encapsulation in liposomes and drug loading capacity of liposomes were calculated with the following equations [24]:


$$\begin{array}{l} Encapsulation\;efficiency\;(EE)\;\%=\frac{total\;amount\;of\;drug-amount\;of\;free\;drug}{total\;amount\;of\;drug}\times 100,\\ Drug\;loading\;(DL)\;\%=\frac{total\;amount\;of\;drug-amount\;of\;free\;drug}{total\;amount\;of\;liposomes}\times 100. \end{array}$$

The quercetin was practically insoluble in water and the small volume of sample in centrifugation may be insufficient for dissolving quercetin. To crosscheck this method, the encapsulation efficiency and the drug loading studies were also replicated using dialysis bag [25]. One milliliter of liposome dispersion (not to be centrifuged) was placed in a dialysis bag (MWCO 12kDa). The dialysis bag including sample was put into the beaker including 200 mL of PBS (pH 7.4). It was kept under stirring at 500 rpm for 24 h. After dialysis, 1 mL of sample was withdrawn and analyzed. The calculation was done according to the abovementioned formula.

### 2.5. In vitro release studies

Quercetin release from liposomes were performed using the dialysis bag method [26]. After the centrifugation, 500 μL of liposomes were placed into the dialysis bag (MWCO 12kDa) following the redispersion of the precipitates with 1 mL of PBS (pH 7.4). The study conditions were set at a stirring speed of 50 rpm, dissolution medium volume 100 mL PBS (pH 7.4) at 37 °C. The samples were withdrawn at predetermined time intervals (10th, 15th and 30th min, and 1st, 2nd, 4th, 6th, and 24th h) to determine the drug amount and replenished with the same medium volume. Experiments were repeated three times. The samples were analyzed spectrophotometrically at 268 nm and 375 nm and expressed as a percentage of the drug release.

### 2.6. Cell viability assay

HeLa human cervical cancer cell lines were used in this study. Cells were cultured in DMEM medium supplemented with 10% fetal bovine serum FBS, 100 U/mL penicillin and 100 μg/mL streptomycin and 2 mM L-glutamine. The cultures were incubated at 37 °C in a humidified atmosphere with 5% CO_2_. The culture medium was replaced with fresh medium every 2 days until reaching suitable confluency of about 90%. All reagents and medium for cell culture studies were purchased from Gibco Company. All experiments were repeated triplicate (n = 3).

The cells except the control cells were treated with different concentrations (1–1000 μg/mL) of quercetin and produced liposomes, all wells were incubated for 12 h, and then washed with PBS and added to 100 μL DMEM. For MTT assay, ten microliters of the MTT (3-(4,5-dimethylthiazol-2-yl)-2,5-diphenyltetrazolium bromide) (Vybrant, Invitrogen) labelling reagent was added to these wells and incubated for 4 h in humidified atmosphere at 37 °C incubator with 5% CO_2_ in air. After the incubation, 100 μL of the SDS buffer was added into each well for solubilization of formazan precipitate. Then absorbance was measured by microplate reader at 570 nm (Epoch, Biotek, USA) and was carried out in triplicate of each assay. Cell viability was calculated as follows [27]:


$$\%\;Cell\;viability=\frac{Abs\;(sample)-Abs\;(blank)}{Abs\;(control)-Abs\;(blank)}\times 100.$$

At the same time, images of HeLa cells before and after treatment of quercetin and produced liposomes were also assessed by inverted microscope attached to camera system (Zeiss Axioivert, Germany).

### 2.7. Colony formation assay

For evaluating colony formation, HeLa cells seeded in twelve well plates (500 cells per well) were treated with quercetin and produced liposomes (10 μg/mL), and cultured in a growth medium for 14 days. The medium was refreshed every three days until incubation was completed. At the end of incubation, each well in the plate was washed with PBS, fixed with cold methanol/acetic acid, stained with 0.5% crystal violet staining solution for 15 min and washed with dH_2_O, respectively. The stained cells were examined with microscope (Zeiss Axioivert, Germany). The number of colonies in each well was counted and analyzed [28].

### 2.8. Hemolysis assay

The freshly obtained blood sample was put into tubes involving EDTA and centrifuged at 1000 g for 15 min. After discarding the supernatant, 10% (w/v) RBC solution was prepared with pellet in PBS (pH 7.4). A total of 100 μL RBC solution and 900 μL quercetin and produced liposomes at the concentration of 10 μg/mL were mixed in Eppendorf and then were incubated at 37 °C for 1 h. Besides 900 μL 0.9% NaCl and dH_2_O were respectively used for negative and positive control. The mixtures were centrifuged at 1000 g for 15 min following incubation and supernatants were spectrophotometrically measured at 540 nm [29]. The experiments were repeated three times. The percentage of hemolysis was calculated as follows:


$$\%\;Hemolysis=\frac{Abs\;(sample)-Abs\;(-control)}{Abs\;(+control)-Abs\;(-control)}\times 100.$$

According to this formula, the negative control was accepted as blank.

### 2.9. AO/PI staining

Propidium iodide (PI) and acridine orange (AO) double staining in cultured HeLa cells were measured to detect apoptosis after the treatment of quercetin and produced liposomes, as previously described [28]. Cells (1 × 10^4^) were cultured in a twelve-well plate and treated with quercetin and produced liposomes (10 μg/mL). After a 12-h incubation, cells were washed with PBS and stained with AO (1 μg/mL) and propidium iodide (1 μg/mL) for 10 min. After discarding the excess dye by washing with PBS repeatedly, images of cells were captured under a fluorescence microscope (Zeiss Axioivert, Germany).

### 2.10. qPCR for gene expression

Total RNA was extracted from 5 × 10^6^ HeLa cells, according to the manufacturer’s instructions (Invitrogen), as previously described [30]. A total of 1 μg RNA was reverse transcribed as the template for cDNA synthesis using high capacity cDNA Reverse Transcription Kit (Applied Biosytem) according to the manufacturer’s instructions. Quantitative real-time PCR was performed for Bax, Bcl-2 and GAPDH using primers. The primers sequence was: Bax: 5′ – GCCCTTTTGCTTCAGGGTTT - 3′ (forward),5′ – TCCAATGTCCAGCCCATGAT - 3′ (reverse); Bcl - 2: 5′ – GACAGAAGATCATGCCGTCC - 3′ (forward), 5′ – GGTACCAATGGCACTTCAAG - 3′ (reverse); GAPDH: 5′ - AGGGCTGCTTTTAACTCTGT - 3′ (forward), 5′ - CCCCACTTGATTTTGGAGGA - 3′ (reverse). One hundred nanograms of cDNA was amplified using SYBR Green PCR Master Mix (Applied Biosytem) on the ABI StepOnePlus detection system, programmed for initial denaturation step, 95 °C for 10 min followed by 40 cycles of denaturation at 95 °C for 15 s; annealing, 1 min at 60 °C; and elongation, 10 s at 72 °C. The amplification results were analyzed using StepOne Software v2.3 (Applied Biosystems, Foster City, CA) and the genes of interest were normalized to the corresponding GAPDH results. Data were expressed as fold induction relative to the control.

### 2.11. Statistical analysis

All experiments were carried out in triplicate and values were expressed as mean ± SD. Statistically significant differences were determined using One-way ANOVA followed by Tukey’s multiple comparison test. Statistically significant differences in all cases were defined as a p-value of < 0.05.

## 3. Results and discussion

### 3.1. Solubility of QC

**Figure 1:**
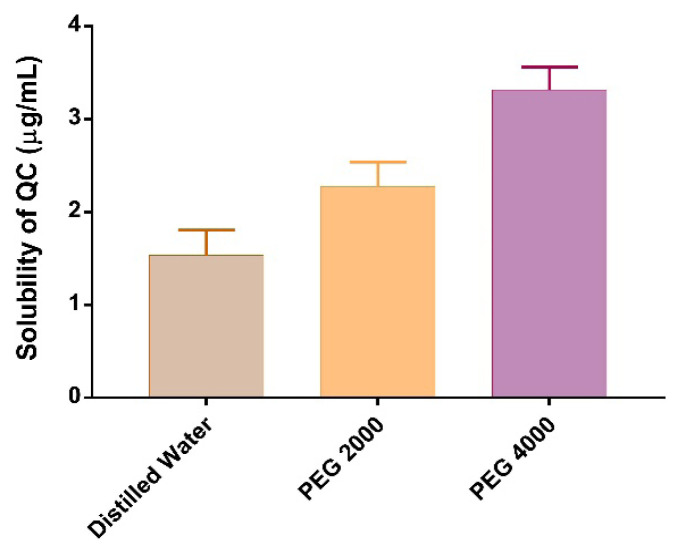
Solubility of QC in distilled water, PEG 2000 and PEG 4000.

The QC solubility test results in various media as pure distilled water and PEG2000 or PEG4000 aqueous solution are presented in Figure 1. The aqueous solubility of anhydrous quercetin was found 1.53 ± 0.27 μg/mL which is in agreement with the value given in the literature [3,31]. Solubilization with surfactants is one of the various techniques to enhance the drug solubility [32]. Adding PEG, a nonionic surfactant, into the solution increased the QC solubility in water, as predictably. PEG2000 increased 1.5-fold of its solubility while 2.2-fold increment occurred in PEG4000 solution. The surfactant molecular weight has a noticeable influence on the drug solubility and the higher molecular weight resulted in the higher solubility. It was also reported that increasing the PEG molecular weight created the better hydrophilicity and water solubility, and increased drug solubility [33]. PEG4000 was used in liposome formulation since it provided better QC solubility.

### 3.2. Particle size and zeta potential measurements

**Figure 2:**
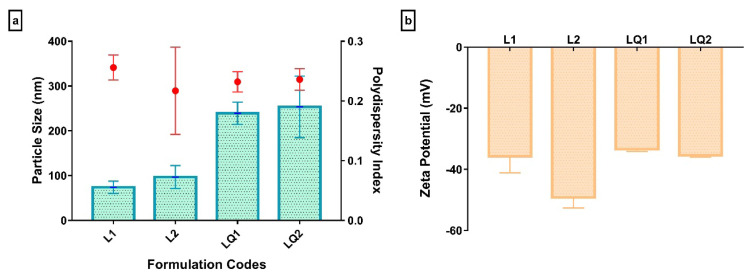
Characterization of liposomes with or without quercetin; a. particle size (bar graph) and polydispersity index (scatter plot) measurement results, b. zeta potential results.

One of the crucial parameters for the characterization of nanocarriers is particle size due to their effect on in vitro behavior and stability, in vivo integrity, retention, cell penetration and consequently intestinal absorption. It is widely known that smaller particles have a greater surface area that ensures convenient solubility. Besides, they might facilitate passive targeting. It is generally accepted that the optimum size is lower than 500 nm for cancer treatment, particularly [34–36]. The particle sizes of produced liposomes were given as hydrodynamic diameter in Figure 2a.

As shown, the particle sizes were varied within the range of 74 ± 14 and 254 ± 69 nm. Although adding polyethylene glycol to liposome structure did not make significant differences in the particle size, it was significantly increased when quercetin was added to the formulations. Two earthly reasons could be stated; (i) the small amount (0.08 mM) of PEG4000 was used in formulations comparing to the amount of quercetin (6.6 mM), (ii) quercetin might have a large surface area due to its cyclic compound while PEG4000 is the straight chain.

The polydispersity index (PDI) is a factor that reflects the particle size distribution and is used to evaluate the colloidal stability of liposomes. The PDI close to 0 implicates the degree of uniformity, while the PDI values higher than 0.4 implicates the heterogeneity, in other words highly polydisperse systems. The PDI’s outstanding value for liposomes indicating a homogenous distribution is considered as 0.3 and below [37, 38]. The PDI of liposomes was < 0.3 of PDI, indicating that they were monodisperse (Figure 2a).

Besides particle size and polydispersity index, the zeta potential plays an essential role in in vitro and in vivo stability. The particles with higher values than ± 30mV are less prone to aggregation or fusion. Moreover, negatively charged liposomes are less phagocytized than positive ones [15,39]. The zeta potentials of liposomes were higher than 30 mV and negatively charged (Figure 2b). These findings implied the positive effects of produced liposomes on stability.

### 3.3. The morphological investigation

**Figure 3:**
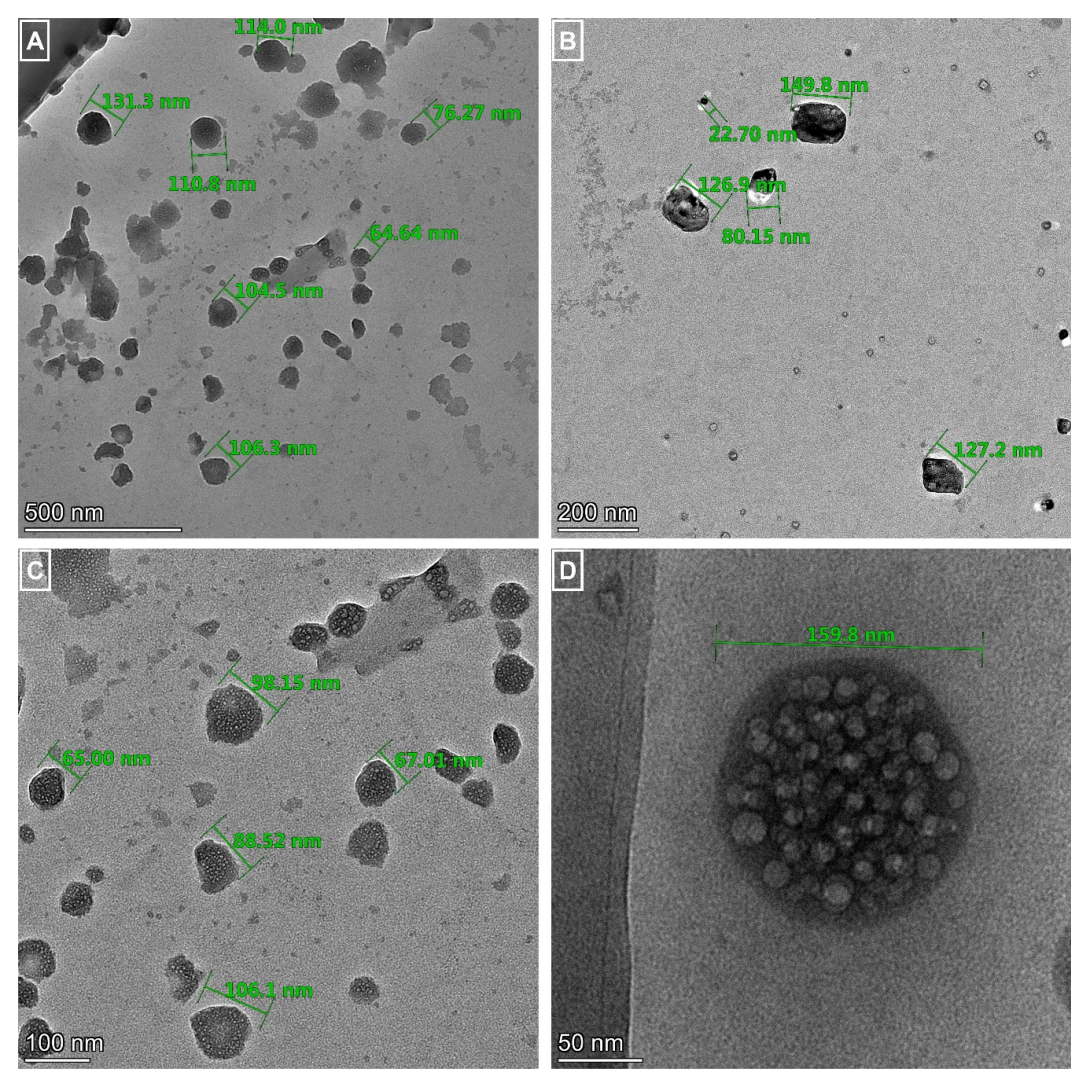
TEM images of quercetin loaded liposomes with different magnifications. Scale bars: A: 500 nm, B: 200 nm, C: 100 nm, and D: 50 nm.

**Figure 4:**
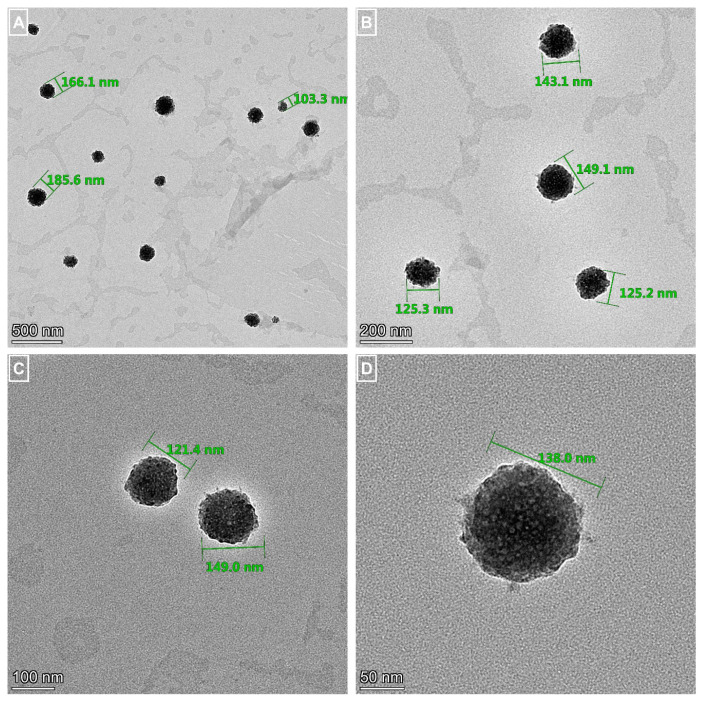
TEM images of quercetin loaded PEGylated liposomes with different magnifications. Scale bars: A: 500 nm, B: 200 nm, C: 100 nm, and D: 50 nm.

TEM images of liposomes were monitored (Figures 3a–3d for QC loaded liposomes, and Figures 4a–4d for QC loaded PEGylated liposomes). TEM images confirmed spherical geometry and the practically monodispersion of prepared samples. Comparing images of liposomes, it was obvious that PEGylation provided the occurrence of more spherical, stable particles and hampered the aggregation tendency of lipids.

### 3.4. Encapsulation efficiency and drug loading capacity

**Figure 5:**
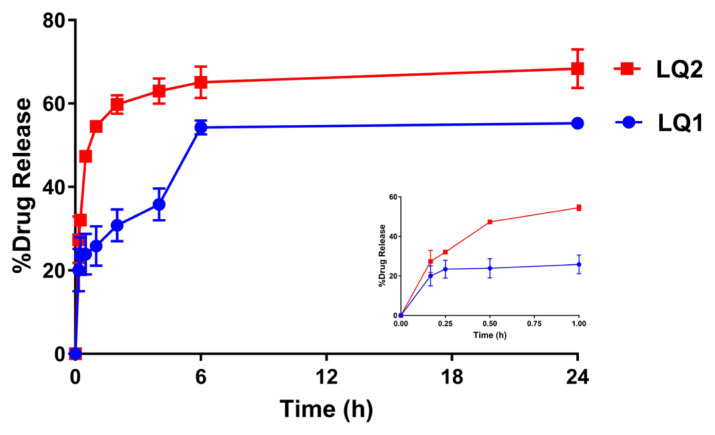
The encapsulation efficiency and drug loading capacity of liposomes with or without PEG coating (LQ1 and LQ2) based on centrifugation or dialysis technique.

The quercetin encapsulation of liposomes was evaluated by UV-VIS spectroscopy according to two indirect methods; centrifugation and dialysis. Centrifugation is mostly preferable method since it is time-saving, easy to apply and both fractions can be kept. However, drugs could be trapped in precipitate in some cases, particularly poorly water-soluble drugs. After centrifugation, a washing process should be done to remove the free drug. That is tricky because of the small volume of diluent. In case of poor separation of free drug from nanocarriers in centrifugation, dialysis bag method was also performed. In this method, higher volume of medium is quite enough to make quercetin soluble. The percentages of EE and DL of quercetin were given in Figure 5.

The EE and DL measurements based on centrifugation were 65.9 ± 4.7% and 17.5 ± 6.0% for quercetin in liposomes, respectively. PEGylation step increased the amount of QC in liposomes up to 87.6 ± 5.6% for EE and 22.2 ± 6.9% for DL. These results could be accounted for by the solubility increasing of QC as a consequence of the surfactant effect of PEG4000. In the dialysis method, similar results were obtained and there were no significant differences between two methods.

### 3.5. In vitro release studies

**Figure 6:**
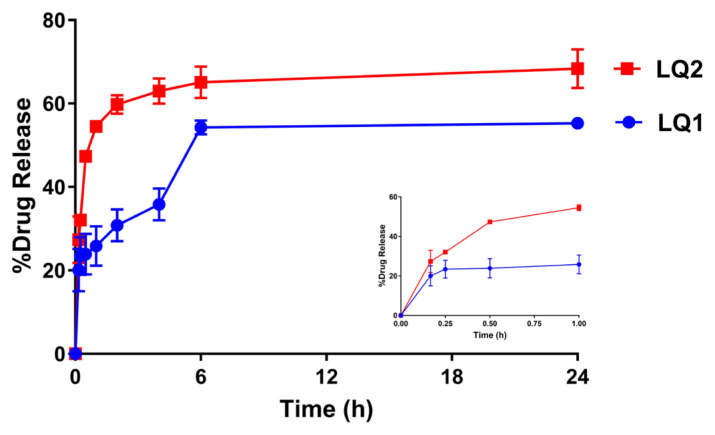
The release profile of quercetin from liposomes (LQ1 and LQ2). The embedded figure which was placed in right below focused the quercetin release on the first 1 hour.

Quercetin release from liposomes were performed through the dialysis bag method [40]. The in vitro release profiles of quercetin loaded liposomes were shown in Figure 6.

The quercetin release from LQ1 liposomes was reached 25.8 ± 4.7% in 1 h (the embedded figure focused on the first 1 hour) while that from LQ2 liposomes were 54.5 ± 1.5%. Since PEG is a hydrophilic polymer, the PEGylation of liposomes might aid the release of quercetin from liposomes. The release was completed in 6 h for both formulations and the total release of QC from LQ2 liposomes was 65.1 ± 3.8%. Not having the 100% of release could be related to the high affinity between lipophilic quercetin and lipophilic nanocarriers.

### 3.6. Cell viability assay

**Figure 7:**
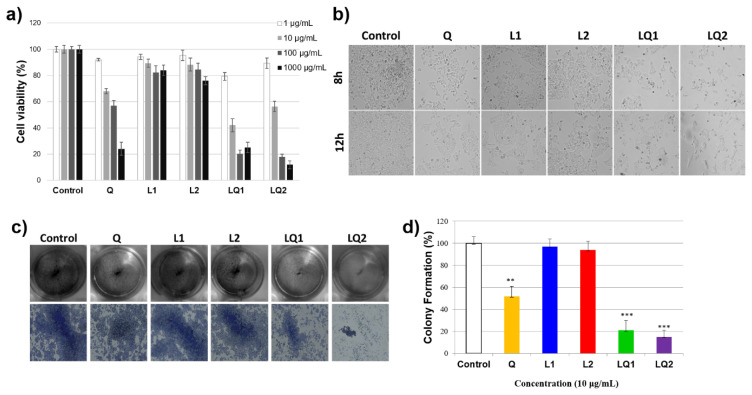
(a) Comparison of cytotoxicity between quercetin and produced liposomes at the concentration of 1–1000 μg/mL, (b) Morphological changes of samples in HeLa cells 8 h and 12 h after treatment. Cellular morphology was observed and captured at 20× magnification using an inverted microscope (c) Colony formation assay in HeLa cells. Cells were treated with 10 μg/mL quercetin and produced liposomes and were cultured for 14 days and were stained with crystal violet. Colonies shown as overview images (up) and detailed images individual colony (down) d) Colony formation ratios were quantitated compared to control groups (**p < 0.01, ***p < 0.001 compared to control in HeLa cells).

Quercetin and all produced liposomes were applied to HeLa cells for 12 hours of incubation. Untreated cells were used as a control representing 100% viability. L1 and L2 (quercetin-free liposomes with and without PEG, respectively) were chosen as negative control groups so that the phospholipid effect, and consequently the physiological tolerability of liposome formulations could be taken into consideration. Phospholipids and sterols like cholesterol are major components of all cell membranes, as well as liposomes. They provide the appropriate structural organization of the membrane bilayer and affect cellular functions like permeability and fluidity [41]. Empty liposomes, L1 and L2, did not cause significant cell death (seen in Figure 7a). There were no statistical differences between control groups and quercetin-free liposomes at low doses, while they showed approximately 80% of cell viability even at their quite high concentration (1 mg/mL). Quercetin showed a concentration-dependent manner on cell survival in the range of 1–1000 μM of concentration. It decreased cell viability from 68% to 57% when increasing its concentration from 10 μg/mL to 100 μg/mL. The IC50 values were found as 88.3 μg/mL for quercetin, 8.1 μg/mL for LQ1, and 16.3 μg/mL for LQ2 in HeLa cells. IC50 value of quercetin was 88.3 μg/mL, which was consistent with previous study [42,43]. Liposomes decreased the IC50 values of quercetin five times compared to LQ2 and 10 times compared to LQ1. It was reported that IC 50 values of quercetin loaded stealth liposomes (with PEG2000) in HeLa cells was 184 μM (equal to 55.8 μg/mL) after 24 h incubation [43]. In this regard, it can be concluded that a much higher therapeutic effect has been achieved with PEGylated liposomal formulations.

On the other hand, a reduction in cell viability of more than 50% was achieved approximately at a dose of 10 μg/mL for LQ1 and LQ2. These results suggest that liposomal quercetin formulations demonstrated a much higher cytotoxic effect on HeLa cells when compared to free quercetin regardless of the presence of PEGylation. However, the effect of quercetin loaded PEGylated liposomes (LQ2) on cell viability became more pronounced at the higher concentration (above 10 μg/mL). After the treatment with the LQ2 formulation at a concentration of 100 μg/mL, the percentage of the surviving cells was 18.0 ± 2.1.

To confirm the MTT results, the morphological changes were investigated by induced by 10 μg/mL of quercetin or produced liposomes with the same concentration. As seen in Figure 7b, L1 and L2 maintained the regular morphology of HeLa cells. There was no significant decrease in viability of the cells like what was observed with the control group. When cells were treated with quercetin, the HeLa cells became round in shape and smaller in size. The number of viable cells and morphological change was quite apparent after LQ1 and LQ2 treatment. The number of living cells was decreased after the L1Q exposure. Moreover, LQ2 caused a significant change in the morphology of HeLa cells with a strong reduction in cell viability. We can conclude that the cytotoxic action of quercetin was boosted in liposomes formulation. On the other hand, no significant decrease in viability of the cells was observed between 8 h-incubation and 12 h-incubation. The possible explanation of it might be related to drug release which was completed at 6 h in L1Q and L2Q formulations.

### 3.7. Colony formation assay

Firstly, we examined the effects of quercetin or produced liposomes on the colony formation ability of HeLa cells. As shown in Figure 7c, when we exposed HeLa cells to quercetin, L1Q and L2Q the number of colony reduced. Mainly in 10 μg/mL doses of L1Q and L2Q, colonies development drastically decreased by 79% and 85%, respectively, compared to the control group (Figure 7d).

### 3.8. Hemolysis assay

**Figure 8:**
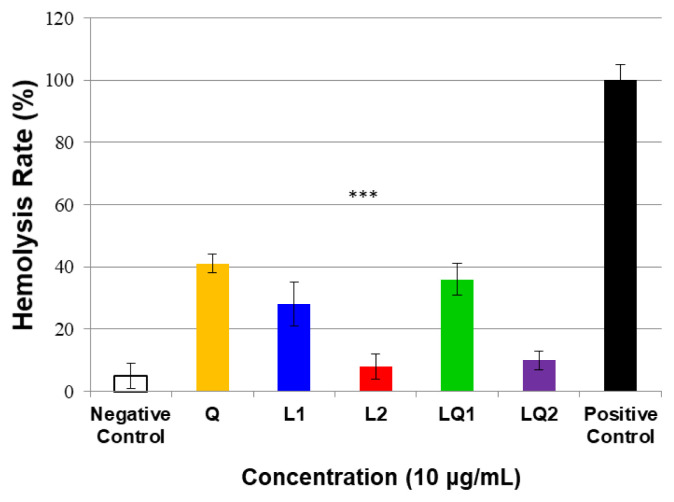
Hemolysis rate (%) after 1 h of incubation at 37 °C with quercetin and all produced liposomes at the concentration of 10 μg/mL. Each value represents the mean and SD (n = 3), ***p < 0.001.

To investigate the safety of produced liposomes and suitability for parenteral use, hemolysis rate of compounds was evaluated, as shown in Figure 8. Hemolysis is defined as a rupture of red blood cells with the release of hemoglobin and other intracellular contents into the plasma [44]. Nonaqueous formulations are known to cause hemolysis. Therefore, evaluating the hemolytic potential of a parenteral formulation is crucial [45]. NaCI, a fully biocompatible agent whose parenteral usage cannot cause hemolysis, was used as a negative control group while dH_2_O, a hemolytic agent, was used as a positive control group. The hemolytic rate of NaCI was 5% and that of dH_2_O was 100%, as expected. Quercetin showed hemolysis of 41%. Generally, hemolysis value of nonhemolytic compounds is below 10% while values above 25% are considered hemolytic [45]. Therefore, quercetin could be considered as a hemolytic agent. The exclusively liposomal formulations of quercetin (LQ1) could not sufficiently improve its potential destructive effect on erythrocyte. Quercetin-free formulation (L1) with lipid structure also caused moderately hemolysis (28%). On the other hand, PEGylation were significantly decreased <10% hemolysis of erythrocytes (8% and 10% for L2 and LQ2, respectively). The presence of polyethylene glycol groups in formulation can conceal the surface of liposomes, and consequently the inhibition of hydrophobic interaction occurs [46]. The stealth liposomes induced a decrease in drug-associated hemolytic toxicity and made the formulation safer and acceptable for parenteral administration.

### 3.9. AO/PI staining

**Figure 9:**
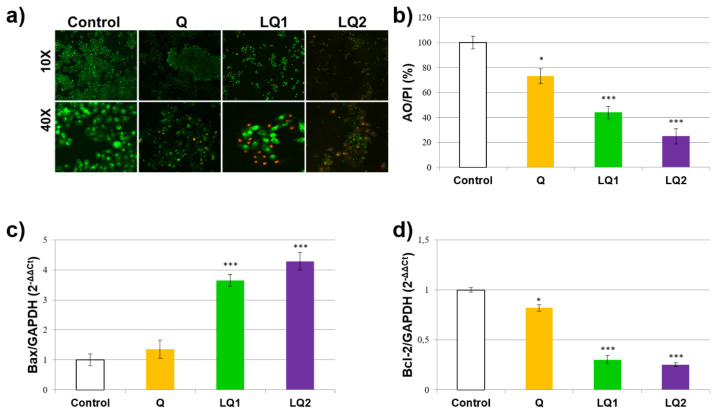
Apoptosis effects of quercetin loaded liposomes and quercetin loaded PEGylated liposomes compared to quercetin; A: cell images after AO/PI staining; green emission refers to live cells while red emission refers to death cells, B: the ratio (%) of the number of viable cells to the number of dead cells according to AO/PI staining, C: the values of Bax protein expressions after treatment and it indicates the proapoptotic activity that induce apoptosis by forming mitochondrial permeability transition pores, D: the values of Bcl-2 protein expressions after treatment and its overexpression protects the cell against apoptosis.

To assess the antitumor potential of quercetin loaded liposomes in human cervical cancer cells, HeLa cells were treated with quercetin, L1Q and L2Q, and AO/PI staining was performed. The AO/PI staining ratio indicates the ratio of the number of viable cells to the number of dead cells. The decrease in the ratio accelerates the apoptosis of the cells (Figure 9a). The AO/PI staining experiments showed a significant decrease (27% higher than the control level) in the quercetin treated HeLa cells. Moreover, AO/PI staining level was decreased in L1Q and L2Q treated groups, with statistical significance (Figure 9b).

### 3.10. qPCR for gene expressions

To investigate the effects of quercetin loaded liposomes treatment on HeLa cell apoptosis, mitochondrial apoptotic gene expression levels were measured such as Bax and Bcl-2. Bax is known as a proapoptotic gene while Bcl-2 is an antiapoptotic gene. Our results showed that quercetin could not influence the mitochondrial apoptotic proteins as Bax. Bax gene expression levels were increased in L1Q and L2Q treated groups compared to control (Figure 9c). On the other hand, Bcl-2 gene expression levels were decreased in quercetin, L1Q and L2Q treated groups compare to control (Figure 9d). These results suggest that the quercetin loaded liposomes treatment could trigger the mitochondrial apoptosis of HeLa cells.

## 4. Conclusion

In the present study, QC loaded PEG4000 modified liposomal formulations were successfully prepared in small size, homogeneously dispersed and spherical shape. Their high encapsulation efficiency (87.6 ± 5.6%) was confirmed by two different methods; centrifugation and dialysis. Cytotoxicity studies demonstrated that liposomal formulation of QC inhibited the cell viability of cervical cancer. PEGylated liposomes remarkably decreased the IC value of free QC. The number of colony diminished in the presence of liposomes, and the PEGylation decreased the colonization tendency of cells to 85%. Formulations were found to be convenient to the parenteral application according to hemolysis assay. The AO/PI staining study showed that the apoptosis of the cells was triggered with these developed nanoliposomal formulations. This result was confirmed with protein expression studies by the occurrence of the increasing mitochondrial apoptotic gene expression levels when cells exposed to the liposomal formulation. This result confirmed the increasing mitochondrial apoptotic gene expression levels in cells exposed to liposomal formulations in protein expression studies. As a result of this study, liposomes as well as PEG4000 increased the solubility of QC and the amount of QC required for therapeutic efficacy could be significantly reduced with this promising drug delivery system.

**Highlights**

- The solubility of quercetin can be increased by adding surfactant. PEG4000 increased quercetin solubility more effectively than PEG2000.
- PEG4000 in liposomal formulation provided the convenience of liposomes for the parenteral application.
- The IC50 value of quercetin in HeLa cells remarkably decreased using PEG4000 modified liposomal quercetin.
- Developed liposomal quercetin at minimal doses induced mitochondrial apoptosis in HeLa cells much effectively than free quercetin.

## Figures and Tables

**Table:** The compositions of liposomes.

Formulation	L1	L2	LQ1	LQ2
**L-α-phosphatidylcholine**	6 mM	6 mM	6 mM	6 mM
**Cholesterol**	3.4 mM	3.4 mM	3.4 mM	3.4 mM
**PEG4000**	-	0.08 mM	-	0.08 mM
**Quercetin**	-	-	6.6 mM	6.6 mM
